# Loneliness in older parents: marital transitions, family and social connections, and separate bedrooms for sleep

**DOI:** 10.1186/s12877-021-02550-x

**Published:** 2021-10-22

**Authors:** Chi Chiao, Wen-Hsu Lin, Yu-Hua Chen, Chin-Chun Yi

**Affiliations:** 1 Institute of Health and Welfare Policy, College of Medicine, National Yang Ming Chiao Tung University, No.155, Sec.2, Linong Street, Taipei City, 112 Taiwan, Republic of China; 2 Institute of Public Health, College of Medicine, National Yang Ming Chiao Tung University, No. 155, Sec. 2, Linong Street, Taipei City, 112 Taiwan, Republic of China; 3 Department of Bio-Industry Communication and Development, National Taiwan University, No. 1, Sec. 4, Roosevelt Road, Taipei City, 10617 Taiwan, Republic of China; 4 Institute of Sociology, Academia Sinica, No. 128, Sec. 2, Academia Road, Nankang, Taipei City, 11529 Taiwan, Republic of China

**Keywords:** Marital transitions, Loneliness, Separate bedrooms for sleep, Taiwan

## Abstract

**Objectives:**

This research innovatively analyzed the marital transitions (i.e., divorce and widowhood) of older Taiwanese parents, their sleep problems and spousal specific characteristics (i.e., separate bedrooms for sleep and marital relationships) as well as their social and family connections, all of which were simultaneously reflected in emotional and social domains of loneliness.

**Methods:**

Data are from 1645 older parents from Northern Taiwan. Loneliness was assessed by a De Jong-Gierveld short scale with emotional and social domains. We conducted multivariate logistic regression to examine the associations of marital transitions and family/social connections regarding sleep problems and psychological well-being with loneliness in social and emotional domains. Besides sleep problems and individual socioeconomic status, we included data on couples’ sleeping arrangements and marital relationships.

**Results:**

Social loneliness was significantly associated with being divorced (AOR = 1.80, 95% CI 1.13–2.86) and living alone (AOR = 1.50, 95% CI 1.02–2.23). In contrast, strong family cohesion and frequent weekly contact with friends were associated with lower social loneliness. Married parents who slept in separate bedrooms were more likely than bed-sharing couples to feel emotional and social loneliness, despite adjusting for their sleep problems. Furthermore, satisfactory spousal relationships significantly decreased the magnitude of associations in the social domain.

**Discussion:**

Our findings support significant associations between loneliness in later life and major marital transitions, family and social connections and sleep problems which differ in social and emotional domains. Independent of relationship satisfaction, separate bedrooms relate to higher risks of emotional loneliness in older adults.

## Introduction

With a rapidly growing population of older adults [1] and international support to improve sleep health over the past few decades [2] loneliness has been associated with sleep problems in older people [3–5], especially in older parents who have experienced major marital transitions. During the coronavirus disease of 2019 (COVID-19) pandemic, loneliness is a growing public health issue [6, 7] found to relate to an increased risk of a higher level of c-reactive protein [8], cardiovascular disease [9], reduced quality of life for individuals and their families [10], and even mortality [11].

Research on loneliness in later life, has often focused on characteristics of individuals and families. Social status variables such as gender, education, work status and family connection predict individual loneliness through their inherent social qualities. Females, less educated older people or those earning low wages are likelier to report feelings of loneliness [12, 13] because loneliness is often considered the psychological embodiment of social isolation [14]. There is ample evidence that family characteristics are associated with older adults’ perceptions of loneliness [11, 15]. The experiences of marital transitions such as divorce and widowhood [16] appear crucial to psychological well-being, and to feelings of loneliness in later life [17–19]. Such marital transitions alter older adults’ perceptions of loneliness as some of their previous social connections become detached, causing isolation from original networks and/or changes in their feelings of loneliness [20].

A related yet separate series of studies found that psychiatric disorders and poor mental health may be related to sleep problems which may be exacerbated in older adults [5, 21]. Cable and colleagues [22] analyzed data gathered from a cohort sample of 3108 older adults from Japan and 7527 older adults from the UK over 3–4 years. Their findings suggest that sleep disturbance correlates with a higher likelihood of the onset of depressive symptomatology at the follow-up assessment in both countries. However, that study is based on a generally older population, and its analyses did not include factors related to the family context regarding life transitions; therefore, additional social and relationship factors, often associated with the mental health of older parents, were not assessed.

An equally important but much less investigated aspect of mental health in older parents is loneliness [15] and even less is its association with sleep behaviors [4]. Therefore, the present study attempts to explain variations in loneliness, from a psychosocial perspective [23], by examining its association with sleep behaviors in older parents and thereby contributes to a research gap. The psychosocial approach focuses on relevant factors including stressors, namely, negative marital transitions, stressful living circumstances, and poor relationship between spouses; the idea of a psychosocial approach includes behavioral and structural components that consider the extent and intensities of associational links and loneliness [12]. This approach highlights the extent to which loneliness is collectively shaped by marital transitions and sleep behaviors through their social and family connections and psychosocial circumstances.

Considering sleep behaviors among older parents as a negotiated social process [24], most married couples are sleep partners, although diverse living arrangements have increasingly been observed among older couples [25]. The investigation on sleep behaviors needs to go beyond the individual perspective [26]. Accordingly, we hypothesize that loneliness in married parents is linked to their sleep arrangements especially considering married couples sleeping in separate bedrooms. Sharing a bed is symbolic of marital status for couples; bed sharing is often seen as “a time of social interaction” [24, 27]. However, a handful of studies have focused on *place* of sleep and loneliness in married couples [28] even few consider the effects of their relationship satisfaction.

Built upon the psychosocial approach [23], the present study explores whether loneliness is associated with major marital transitions, namely divorce and widowhood, and sleep problems (i.e., subjective sleep quality, sleep disturbance, use of sleep medicine, and daytime dysfunction). Loneliness includes both emotional and social domains [29–31]; we assess how social and family connections are related to the aforementioned associations in older parents, independent of other psychological well-being indicators (i.e., depressive symptoms, self-esteem, and fulfillment) as well as their health status (i.e., subjective self-rated health of respondents and to their spouse). We further explore differences in loneliness across each domain when considering couple related characteristics such as separate bedrooms and spousal relationships in married parents.

## Methods

### Data

The present study used data from a large-scale survey of the Taiwan Youth Project (TYP). The TYP used a multi-stage random sampling frame to obtain a cohort sample of school-based students and their parents in Northern Taiwan. The detailed sampling design and data collection procedures have been described in previous studies [32]. The surveys began in 2000, with follow-ups in 2002, 2005, 2007, and 2019; however, the TYP did not collect data on sleep problems, sleep arrangements and loneliness in the parent sample until 2019, as it was around the time that parents were approaching older adulthood. Face-to-face interviews were conducted between January 2019 and December 2020 in three administrative divisions (Taipei City, New Taipei City and Yi-Lan County) of Northern Taiwan.

For the purpose of this study, we analyzed the 2019–2020 survey data of older parents (50 to 75 years old with average age of 62 years old). Excluded from the current analysis were parents with incomplete major explanatory variables (*N* = 17), which yielded a final sample of 1645 parents with 1292 currently married. This sample size is sufficient to reach a power of 0.90 [33]. TYP data are publicly available and can be used for research with the approval of Academia Sinica in Taiwan (http://www.typ.sinica.edu.tw). All TYP participants gave informed written consent at the start of their interviews. The present study protocol was approved by the Research Ethics Committee of National Yang-Ming University (Taipei, Taiwan) (IRB Number: YM109021E).

### Measures

Loneliness is assessed through self-reported data from a 6-item de Jong-Gierveld short scale (DJGS) [18, 29], which includes two dimensions: emotional loneliness and social loneliness [31]. Each item was rated on a three-point scale, indicating the severity of each situation. The use of this 6-item DJGS has been validated in studies of older Chinese people in Hong Kong [34]. Preliminary analyses that tested internal consistency revealed good reliability (α = 0.75) on the three items of social domain but poor reliability (α = 0.40) on the three items of emotional domain. Rather than the subscale of emotional loneliness, we selected an item that represented emotional loneliness [18, 29, 35]. Subjects were asked whether they had felt lonely during the past 2 weeks and responses were dichotomized: “feelings of being lonely” (coded as 1), including slightly serious, serious, and very serious feelings of loneliness, while “no feelings of being lonely” was coded as 0. For simplicity, we used the same coding scheme to assess social loneliness. Each of the three items of social loneliness was first recoded into two categories: not lonely (coded 0) or lonely/extremely lonely (coded 1). The total score on this domain ranged from 0 to 3. The score was further dichotomized into not lonely (0 to 2) or extremely lonely (3) based on suggestions from prior studies [29].

Social and family connection measures in the current study underscore network connection and the objective aspect of loneliness [11]. According to the research aims, the first part of the main explanatory variables included marital status, living alone (binary item), family cohesion, social contact with friends (from never to always: 0 to 3), and household size (number of people in the household). Marital status was assessed by the question, “What is your current marital status?” The responses were categorized into currently married, widowed, divorced, or others. Family cohesion was measured using 4 items of the family cohesion scale [36, 37]. Items on this measure assess the extent of participating in family-related activities, receiving family comfort, being involved in family discussion, and relying on my family. A higher score represents stronger cohesion; this measure showed good reliability (Cronbach alpha = 0.88).

Sleep problems were measured by the Pittsburgh Sleep Quality Index (PSQI) [38] that comprises four components in this study: sleep quality, sleep disturbance, use of sleep medicine and daytime dysfunction. The score on each component ranged from 0 to 3; higher scores represented poorer sleep. This scale has both acceptable reliability [39] and validity [40], and has previously been used in Taiwan [41].

The present study hypothesized that spousal characteristics are related to their loneliness [42]. The second part of main explanatory variables focused on spousal factors, namely separate bedrooms, sex more than once per month, perception of spousal poor health, marital duration (in years), and marital relationships (i.e., satisfying spousal relationship and intimate partner violence and having a Facebook post). Separate bedrooms were measured by the presence of one of four types of sleeping arrangement, namely bed sharing, different beds in the same bedroom, separate bedrooms, or unknown status. Information about *having sex more than once per month* was obtained by asking how often you had sex with your spouse/partner. Then, marital relationships were measured by three indicators: satisfying spousal relationship (from very dissatisfied to very satisfied: 0 to 3), intimate partner violence (total score of five types of violence; range from 0 to 5), and ever had a Facebook post (yes vs. no).

Psychological well-being includes three self-reported measures, namely, depressive symptomatology, self-esteem, and feeling fulfilled, as well as self-rated health status. Depressive symptomatology used an 11-item version of the Symptom Checklist-90 Revised (SCL-90-R) [43, 44]. Preliminary factor analyses on the 11 items revealed two factors, depressive and somatic domains (results not shown). The total score of the depressive domain ranged from 5 to 25 and yielded good internal consistency and reliability (α = 0.76). The other six SCL-90-R items in the somatic domain had a total score ranging from 6 to 30 with good internal consistency and reliability across the waves (α = 0.75). Higher scores represented higher levels of depressive symptoms within each domain. Self-esteem was assessed by 6 items of the Rosenberg’s Self-Esteem Scale [45]. The total score ranged from 6 to 24 and yielded good reliability (Cronbach’s α = 0.79). Higher scores represented higher levels of self-esteem. A feeling of fulfillment was measured by the question, “In general, do you feel fulfilled?” on a 5-point scale (0 = very unfulfilled, 4 = very fulfilled) [46]. Self-rated health is a general health rating of respondents and the perception to their spousal health status, which involves the questions: “In general, would you rate your/your spouse’s current health as excellent, very good, good, fair or poor?” and grouped into poor (fair/poor) vs. good (excellent/very good/good) categories.

Several covariates are included in two categories: *demographic* (sex and age) and *socioeconomic status* (SES) (educational attainment, employment status and perceived economic strain). These covariates were significantly related to sleep problems and loneliness in older adults [12, 13].

### Analytical strategy

To assess the research aims, the investigation employs a 2-part model analysis to explore how feelings of loneliness were associated with sleep problems and social connection, namely divorce, widowhood, living alone, family cohesion, weekly contact with friends and household size among 1645 older parents. We highlighted the sample for marital relationships (*N* = 1294) and assessed how bed-sharing and spousal related characteristics were related to loneliness in both emotional and social domains. We first employed logistic regression techniques to examine the likelihood of feelings of loneliness, including emotional and social domains regarding social and family connections and sleep problems among the 1645 older parents, independent of other aspects of psychological well-being (depressive symptoms, self-esteem, fulfillment, and self-rated health).

As a second step, logistic regression models focused on the married sample of 1259 older parents to study the associations between bed-sharing and the likelihood of reporting loneliness. In addition to sleep problems, health status and spousal relationships were also important to a feeling of loneliness. We conducted a progressive modeling strategy; Model 1 estimated the association of separate bedrooms for each domain of loneliness, simultaneously considering sleep problems, sexual frequency, self-rated health status (both respondents and their perception to their spouse) and individual covariates. Moreover, Model 2 added variables related to spousal relationship, namely spousal relationship satisfaction, intimate partner violence and having a post on Facebook. In this second part of the analysis, we explored a question that had rarely been asked: are older parents who sleep in separate bedrooms likelier to experience feelings of loneliness? All models were analyzed using STATA 16.0 [47].

## Results

Table 1 presents descriptive statistics for the study sample. Participant’s average age was 62 years old with a range from 50 to 75, and 67% were females, while 10% reported experiencing economic strain. About four fifths (79%) of the sample were currently married, 14% were widowed and 6% were divorced. One-tenth of the sample currently lived alone. The mean score for self-esteem was 18.08 with a standard deviation (SD) of 2.27 and a range between 8 and 24. About less than half (47%) reported poor health; the mean score for fulfillment was 2.93 (SD = 0.74) with a range between 0 and 4. The average score for subjective sleep quality was 1.20 (SD = 0.70) with a range from 0 to 3. About one-fifth (19%) of the married couples slept in separate bedrooms. Among the participants investigated in this study, 12% reported experiencing emotional loneliness; 30% experienced social loneliness.

**Table 1:** Descriptive characteristics of the total sample and the married sample of young elderly parents [percent or mean (SD)]

	Percent or mean (SD)	
Variable	Total sample	The married sample
*Social and family connection variables*		
Marital status (%)		
Married	78.54	
Widowed	13.98	
Divorced	6.44	
Others	1.03	
Living alone (%)	9.91	4.72
Family cohesion (range: 0–12)	9.60 (2.16)	9.68 (2.10)
Weekly contact with friends (range: 0–3)	3.71 (1.78)	1.71 (0.97)
Household size (range: 1–15)	1.68 (1.00)	3.89 (1.77)
*Psychological well-being*		
Depressive symptoms		
Depressive domain (range: 5–25)	5.81 (1.74)	5.74 (1.64)
Somatic domain (range: 6–30)	8.37 (3.01)	8.26 (2.92)
Self-esteem (range: 8–24)	18.08 (2.27)	18.14 (2.27)
Feeling of fulfillment (range: 0–4)	2.93 (0.74)	2.98 (0.73)
Perceived poor health (%)	47.11	46.36
*Covariates*		
Age (range: 50–75)	62.03 (4.23)	62.03 (4.10)
Male (%)	32.52	35.68
Education attainment (%)		
Primary attainment	44.01	41.80
High school education	43.16	44.58
College and above	12.83	13.62
Currently full-time employed (%)	35.26	34.37
Perceived economic strain (%)	10.33	9.21
Sleep problems		
Subjective sleep quality (range: 0–3)	1.20 (0.70)	1.18 (0.69)
Sleep disturbance (range: 0–3)	0.82 (0.46)	0.82 (0.44)
Use of sleep medicine (range: 0–3)	0.29 (0.83)	0.27 (0.80)
Daytime dysfunction (range: 0–3)	0.21 (0.44)	0.20 (0.42)
Spousal characteristics		
Separate bedrooms for sleep		
Yes		19.12
Couple bed-sharing		55.03
Different beds in the same bedroom		8.67
Unknown		17.18
Had a sex (more than once per month)		25.85
Perception of spousal poor health (%)		47.02
Marital duration in years		35.87 (4.66)
Satisfying spousal relationship (range: 0–3)		2.37 (0.62)
Intimate partner violence (range: 0–3)		0.67 (0.68)
Had a post on Facebook		25.39
Loneliness		
Emotional domain	11.67	9.21
Social domain	30.33	27.94
*N*	1645	1292

*SD* standard deviation

### Associations between social/family connection, sleep problems and loneliness

To investigate two domains of feelings of loneliness, the results from logistic regression models using the total sample are presented in Table 2. This demonstrates that social and family connection and sleep problems were associated with the likelihood of being in a particular domain, even when considering psychological well-being and covariates. Statistical significance was set at a *p*-value lower than 0.10 due to the small sample size of the emotional loners (*N* = 195). In this section, we discuss only statistically significant effects. With emotional loneliness, older parents who were widowed were likelier to experience emotional loneliness than the married group (AOR = 3.52, 95% CI 2.17–5.73). In contrast, an increase in family cohesion was associated with a decrease in feelings of loneliness (AOR = 0.88, 95% CI 0.81–0.97). Daytime dysfunction is significantly related to feelings of emotional loneliness (AOR = 1.82, 95% CI 1.23–2.70).

**Table 2:** Multivariate logistic regression results for feelings of loneliness in the emotional and social domains among young elderly parents, *N* = 1645

	Loneliness: AOR (95% CI)	
	Emotional Domain	Social Domain
*Social and family connection variables*		
Marital status (ref = Married)		
Divorced	1.16 (0.52, 2.57)	1.80 (1.13, 2.86)^*^
Widowed	3.52 (2.17, 5.73)^**^	1.11 (0.79, 1.58)
Others	3.51 (0.82, 14.97)^§^	1.85 (0.61, 5.66)
Living alone	1.10 (0.59, 2.04)	1.50 (1.02, 2.23)^*^
Family cohesion	0.88 (0.81, 0.97)^**^	0.79 (0.75, 0.84)^**^
Weekly contact with friends	0.92 (0.75, 1.13)	0.81 (0.71, 0.92)^**^
Household size	0.91 (0.81, 1.03)	0.95 (0.88, 1.02)
Sleep problems		
Subjective sleep quality	1.04 (0.76, 1.42)	1.31 (1.08, 1.60)^*^
Sleep disturbance	1.17 (0.73, 1.87)	1.03 (0.76, 1.39)
Use of sleep medicine	0.91 (0.73, 1.15)	0.80 (0.69, 0.94)^**^
Daytime dysfunction	1.82 (1.23, 2.70)^**^	1.07 (0.80, 1.43)
*Psychological well-being*		
Depressive symptoms		
Depressive domain	1.85 (1.64, 2.09)^**^	1.06 (0.98, 1.16)
Somatic domain	0.98 (0.92, 1.06)	0.97 (0.92, 1.02)
Self-esteem	0.95 (0.87, 1.04)	0.92 (0.87, 0.98)^**^
Feeling of fulfillment	0.78 (0.59, 1.03)^§^	0.62 (0.51, 0.74)^**^
Perceived poor health (ref = Good)	1.16 (0.76, 1.77)	1.39 (1.08, 1.79)^*^
*Covariates*		
Male (ref = Female)	2.38 (1.52, 3.73)^**^	1.11 (0.84, 1.46)
Age	0.96 (0.91, 1.01)^§^	1.02 (0.99, 1.05)
Education (ref = Primary education)		
High school	1.14 (0.74, 1.75)	1.27 (0.97, 1.66)^§^
College and above	1.51 (0.78, 2.90)	2.17 (1.46, 3.20)^**^
Perceived economic strain (ref = Sufficient)	1.34 (0.80, 2.32)	1.29 (0.88, 1.89)

*Abbreviations*: *AOR* adjusted odds ratio, *CI* confidence interval

^§^*p* < 0.10; ^*^p < 0.05; ^**^p < 0.01

Moreover, social and family connections were significant factors of social loneliness. Older parents who were likelier to report a feeling of social loneliness were divorced (AOR = 1.80, 95% CI: 1.13–2.86) or living alone (AOR = 1.17, 95% CI: 1.07–1.26). In contrast, strong family cohesion and frequent weekly contact with friends were associated with lower likelihoods of reports about feelings of social loneliness. Poor subjective sleep quality is significantly associated with higher incidences of reporting social loneliness (AOR = 3.02, 95% CI 1.21–7.56). The use of sleep medicine was associated with decreased possibilities of being social loners (AOR = 0.80, 95% CI 0.69–0.94).

Furthermore, as a measure of psychological well-being, a higher level of depressive symptomatology in the depressive domain is associated with an increased likelihood of feeling emotional loneliness (AOR = 1.85, 95% CI 1.64–2.09). This significant result was observed, even after adjusting for various covariates. An increase in self-esteem and a feeling of fulfillment is related to lower chances of being social loners; perceived poor health is associated with an increased incidence of reporting social loneliness (AOR = 1.39, 95% CI 1.08–1.79).

### Separate bedrooms for sleep and loneliness among older married parents

With an increase in grey divorce [48], the maintenance of a stable marital relationship seems to be a major contributing factor for older parents; as a result, the sample contains those currently married. Table 3 shows a progressive strategy to bring attention to a possible moderating effect of spousal characteristics on the association between separate bedrooms and loneliness in both emotional and social domains. In addition to sleep arrangements, self-rated health, and perception of spousal poor health, spousal-related measures also consisted of sexual activity, marital duration, satisfying spousal relationship, intimate partner violence, and a Facebook post.

**Table 3:** Multivariate logistic regression results for sleep divorce associated with feelings of loneliness in the emotional and social domains among young elderly parents who were currently married, *N* = 1259

	Loneliness: AOR (95% CI)			
	Emotional Domain		Social Domain	
Covariate	Model 1	Model 2	Model 1	Model 2
Separate bedrooms for sleep (ref = Couple bed-sharing)				
Yes	2.21 (1.36, 3.58)^**^	1.85 (1.12, 3.09)^*^	1.37 (0.99, 1.91)^§^	1.24 (0.88, 1.75)
Different beds in the same bedroom	0.95 (0.43, 2.11)	0.94 (0.42, 2.10)	0.94 (0.59, 1.50)	0.92 (0.57, 1.47)
Unknown	0.63 (0.31, 1.29)	0.70 (0.34, 1.44)	0.43 (0.28, 0.67)^**^	0.44 (0.28, 0.68)^**^
Sleep problems				
Subjective sleep quality	1.05 (0.75, 1.47)	0.98 (0.70, 1.39)	1.52 (1.22, 1.88)^**^	1.44 (1.16, 1.79)^**^
Sleep disturbance	2.00 (1.24, 3.21)^**^	1.99 (1.22, 3.24)^**^	1.13 (0.82, 1.56)	1.11 (0.80, 1.53)
Use of sleep medicine	1.00 (0.79, 1.27)	1.07 (0.84, 1.36)	0.83 (0.70, 0.99)^*^	0.86 (0.72, 1.02)^§^
Daytime dysfunction	2.90 (1.97, 4.27)^**^	2.72 (1.83, 4.06)^**^	1.36 (1.003, 1.85)^*^	1.29 (0.94, 1.77)
Perceived poor health (ref = Good)	1.29 (0.75, 2.24)	1.33 (0.76, 2.32)	1.28 (0.92, 1.77)	1.31 (0.94, 1.82)
Perception of spousal poor health (ref = Good)	1.88 (1.11, 3.20)^*^	1.58 (0.92, 2.72)^§^	1.65 (1.20, 2.27)^**^	1.49 (1.08, 2.06)^*^
Had a sex (more than once per month) (ref = No)	0.87 (0.51, 1.47)	0.93 (0.54, 1.61)	0.75 (0.54, 1.02)^§^	0.81 (0.59, 1.12)
Satisfying spousal relationship (ref = Not satisfied)		0.64 (0.45, 0.92)^*^		0.63 (0.50, 0.80)^**^
Intimate partner violence		1.44 (1.06, 1.97)^*^		1.17 (0.95, 1.44)
Had a post on Facebook (ref = No)		0.61 (0.35, 1.05)^§^		0.67 (0.49, 0.93)^*^
Male (ref = Female)	1.73 (1.07, 2.79)^*^	2.06 (1.25, 3.39)^**^	1.20 (0.89, 1.62)	1.37 (1.01, 1.87)^*^
Age	0.95 (0.89, 1.01)	0.94 (0.88, 1.004)^§^	1.02 (0.98, 1.06)	1.01 (0.97, 1.05)
Marital duration in years	1.04 (0.98, 1.10)	1.04 (0.98, 1.11)	0.99 (0.96, 1.02)	0.99 (0.96, 1.02)
Education (ref = Primary education)				
High school	1.02 (0.63, 1.64)	1.06 (0.65, 1.74)	1.09 (0.81, 1.48)	1.15 (0.85, 1.57)
College and above	1.42 (0.74, 2.75)	1.64 (0.84, 3.21)	1.62 (1.08, 2.44)^*^	1.80 (1.18, 2.73)^**^
Perceived economic strain (ref = Sufficient)	2.53 (1.40, 4.59)^**^	2.26 (1.23, 4.15)^**^	1.90 (1.22, 2.95)^**^	1.70 (1.08, 2.66)^*^

*Abbreviations*: *AOR* adjusted odds ratio, *CI* confidence interval

^§^*p* < 0.10; ^*^p < 0.05; ^**^p < 0.01

In Model 1, the results indicated that married couples who slept in separate bedrooms were likelier to report feelings of loneliness in both emotional (AOR = 2.21, 95% CI: 1.36–3.58) and social (AOR = 1.37 95% CI: 0.99–1.91) domains, compared to the bed-sharing group. Model 2 adjusts the association between sleep arrangements and loneliness for three measures of spouse-specific variables. This inclusion reduced the coefficient results for bed-sharing in both domains of loneliness; thus, the associations remain significant in the emotional but not in the social domain.

In addition, three measures of spouse-specific variables are significantly related to loneliness in both emotional and social domains. Older married parents with a satisfied spousal relationship and a Facebook post are less likely to report loneliness in both domains. Intimate partner violence is associated with an increased likelihood of emotional loneliness (AOR = 1.44; 95% CI 1.06–1.97) but is not significantly related to social loneliness. Perception of spousal poor health was found to be associated with increased odds of loneliness in both emotional (AOR = 1.58, 95% CI: 0.92–2.72) and social (AOR = 1.49, 95% CI: 1.08–2.06) domains, although individual self-rated health was not significantly associated with either emotional or social loneliness. Perceived economic strain was found to be significantly related to higher odds of loneliness in both emotional (AOR = 2.26, 95% CI: 1.23–4.15) and social (AOR = 1.70, 95% CI: 1.80–2.66) domains. Chances of being an emotional and social loner increased with the participant of the married sample being male and experiencing economic strain.

## Discussion

The present study yields several important findings. First, the results support the relationship between family/social connections and loneliness [17, 18, 20] by showing a significant association between marital transitions, namely divorce and widowhood, and loneliness among older parents and this relationship persisted, even after considering psychological well-being. Second, the results are consistent with the hypothesis that there is a significant association between sleep problems and loneliness [4]. The analyses innovatively demonstrate that couples sleeping in separate bedrooms related only to emotional loneliness, and not to social loneliness; with this finding it should be noted that social constructions of sleep behaviors may shape various domains of loneliness, differently. Spousal relationships moderate the sleep-loneliness association. Finally, loneliness in both emotional and social domains was found to be more severe among older parents who perceived economic strain [12, 13]. Interestingly, a feeling of loneliness in both emotional and social domains was more severe among older fathers. Moreover, probably due to the selection issue of maintaining marital union, the aforementioned associations are only significant in married couples, rather than in the total sample.

Widowhood was related to an increased risk of loneliness in the emotional rather than in the social domain; being divorced and living alone were both more related to an increased risk of social loneliness rather than to emotional loneliness, regardless of gender. These results emphasize social and family connections, namely weekly contact with friends and family cohesion, on the social domain of loneliness. Emotional loners are more common among widowed parents when widowhood does not produce a dramatic change in social and family connections [4, 13]. However, being divorced and living alone seem to bring a significant re-arrangement in family and social connections, which, in turn, is associated with the risks of social loneliness. Taiwan, similar to other East Asian countries such as Japan and South Korea, has a long-standing cultural background in Confucian ideology. This ideology emphasizes a popular Chinese slogan “*jia he wan shi xing*” (family harmony serves as the foundation for success on all occasions). Considering the cohort background of these older parents, being divorced, and older parents living alone were often referred to as non-*traditional* events, which re-shaped family and social connections and even their social loneliness. Along this *tradition*, bed-sharing is symbolic of marriage but place of sleep among couples is private and less socially valued [24]. Our results suggest that sleep arrangements in married couples, similar to widowhood in older parents, maintained family and social connections but were linked to emotional loneliness in a non-Western society.

The considerable influence of spousal relationships on loneliness, regardless of sleep arrangements, illustrates the significance of a satisfactory spousal relationship, intimate partner violence and positing life in social media. While the satisfaction of spousal relationships and separate bedrooms for sleep influence and interact with each other in complex ways [24], our results indicate that satisfying spousal relationships and posting about one’s life on Facebook largely explain the association between separate bedrooms and social loneliness, even adjusting for general health status of individuals and their perception of spousal poor health. In contrast, satisfaction with spousal relationships, intimate partner violence and separate bedrooms have independent associations with emotional loneliness. This implies that among these married couples, satisfying spousal relationships or separate bedrooms were associated with lower odds of emotional loneliness, again partly due to reverse association (married couples who reported higher levels of emotional loneliness are likely to have lower levels of satisfaction in their spousal relationships and were likelier to sleep in separate bedrooms).

This study addressed a less explored subject with regard to the linkage between separate bedrooms for sleep in married couples and their loneliness, which may be differentiated into their subjective health rating and perception to their spousal health status [26–28]. In addition to spousal relationships, separate bedrooms for sleep may also be associated with health problem in the married couples. Our findings show that perception of spousal poor health, rather than individual subjective health rating, is significantly related to increased risks of loneliness in both emotional and social domains. Additional analyses on perception of spousal poor health in this married sample also find that 59.35% of those who slept in separate bedrooms report their perception of spousal poor health in comparison to 41.75% with bed-sharing. Not only are separate bedrooms for sleep a significant factor of their loneliness, their perception of spousal health status also has a significant effect.

Our results concur with research on the associations between sleep problems and loneliness in the older population [4, 12] and the findings extend such results by showing there is a significant association between poor sleep quality and social loneliness among these older parents currently in marital union. Consistent with the clinical evidence, poor sleep is a contributing factor to psychotic experiences [5, 21, 22]. The present study further demonstrates that subjective poor sleep quality is related to a feeling of social loneliness, and daytime dysfunction is associated with emotional loneliness, in an empirical manner. This may be partly because various domains of loneliness represent not only their link to psychotic experiences, but also various sleep indicators contribute differently to the psychotic experiences, which may depend on where participants live and work. For instance, sleep disturbance is a risk factor of emotional loneliness only in married couples. Future studies on this issue are needed and they need to be conducted from a multi-dimensional perspective of sleep and loneliness and be marital context-sensitive.

This research innovatively analyzed marital transitions of older parents, their sleep problems and spousal specific characteristics, namely separate bedrooms of sleep and spousal relationships, all of which were reflected in two domains of loneliness simultaneously. Nevertheless, there are several limitations to our study. First, the sample included 17% of married parents who had an unknown status for sleep arrangements. If the subgroup with unknown bed-sharing status was excluded, this would significantly decrease the sample size and consequently the statistical power of the multivariate analyses. However, in the present investigation, bed-sharing status was not found to be significantly associated with social loneliness but appeared to be a risk factor of emotional loneliness. It was possible to further assess the differences in marital duration and loneliness between the known and unknown bed-sharing groups (the results are not tabled). The analyses indicated that this unknown group had the longest duration of marriage and the lowest risk of reporting emotional and social loneliness. As a result, inclusion of the unknown bed-sharing status does not seem to be biased towards the findings. Second, in a Taiwanese context, our results may suffer from an under-reporting of couples sleeping in separate bedrooms, commonly called *sleep divorce* by media portrayals, due to social desirability/undesirability issues. However, it should be noted that not only were extensive efforts made to retain samples in the process, our long-term cooperative relationship with the participants and their children since the initial assessment in 2000 helped to gain participants’ trust. Third, the TYP dataset is based on the self-reported recall of sleep problems and loneliness, which raises the issue of recall bias. Fourth, while prior research has suggested the importance of social networks and support on the status of loneliness (namely network size and providing/receiving social support), such information was not available to the present investigation. Finally, like any cross-sectional data analysis, our study has limitations to disentangle or establish the causal links suggested herein. However, this theory-based study using the logistic regression model does provide important insights; it identifies independent associations related to sleep problems and two domains of loneliness of older parents, independent of psychological well-being.

Despite the limitations, to our knowledge, only a handful of studies have used sleep information from survey data in Asian populations to address two domains of loneliness among older parents. Regardless of psychological well-being, specific sleep indicators, such as sleep quality, sleep disturbance and sleeping in separate bedrooms, vary in their associations with two domains of loneliness. Social and family connection in general, and family cohesion and particularly weekly friend contacts, appear to protect older parents from feelings of loneliness. Future research is needed to better understand distinct domains of loneliness in inter-generational and social network contexts.

## Data Availability

The Taiwan Youth Project (TYP) dataset supporting the conclusions of this article was made available under approval from Institute of Sociology, *Academia Sinica* at http://www.icpsr.umich.edu/icpsrweb/NACDA/studies/3792.
